# Reconstructing a disambiguation sequence that forms perceptual memory of multistable displays via reverse correlation method: Bias onset perception but gently

**DOI:** 10.1167/jov.23.3.10

**Published:** 2023-03-17

**Authors:** Alexander Pastukhov, Lisa Koßmann, Claus-Christian Carbon

**Affiliations:** 1 Department of General Psychology and Methodology, University of Bamberg, Bamberg, Bavaria, Germany; 2 Research Group EPÆG (Ergonomics, Psychological Æsthetics, Gestalt), Bamberg, Bavaria, Germany; 3 Department of General Psychology and Methodology, University of Bamberg, Bamberg, Bavaria, Germany; 4 Research Group EPÆG (Ergonomics, Psychological Æsthetics, Gestalt), Bamberg, Bavaria, Germany; 5 Department of General Psychology and Methodology, University of Bamberg, Bamberg, Bavaria, Germany; 6 Research Group EPÆG (Ergonomics, Psychological Æsthetics, Gestalt), Bamberg, Bavaria, Germany

**Keywords:** perceptual memory, reverse correlation, multistability, kinetic depth effect, structure from motion, psychophysics

## Abstract

When multistable displays are presented intermittently with long blank intervals, their onset perception is determined by perceptual memory of multistable displays. We investigated when and how it is formed using a reverse correlation method and bistable kinetic depth effect displays. Each experimental block consisted of interleaved fully ambiguous probe and exogenously disambiguated prime displays. The purpose of the former was to “read out” the perceptual memory, whereas the latter contained purely random disambiguation sequences that were presented at the beginning of the prime display, throughout the entire presentation, or at the beginning and the end of the presentation. For each experiment and condition, we selected a subset of trials with disambiguation sequences that led to a change in perception of either the prime itself (sequences that modified perception) or the following fully ambiguous probe (sequences that modified perceptual memory). We estimated average disambiguation sequences for each participant using additive linear models. We found that an optimal sequence started at the onset with a moderate disambiguation against the previously dominant state (dominant perception for the previous probe) that gradually reduced until the display is fully ambiguous. We also show that the same sequence leads to an altered perception of the prime, indicating that perception and perceptual memory form at the same time. We suggest that perceptual memory is a consequence of an earlier evidence accumulation process and is informative about how the visual system treated ambiguity in the past rather than how it anticipates an uncertain future.

## Introduction

Our visual system computes a singular representation of an outside world that we can act upon from noisy and intrinsically ambiguous sensory inputs (Yuille & Kersten, 2006). Sometimes sensory inputs are ambiguous in a balanced way so that two (or more) perceptual interpretations are equally likely. When viewed continuously, these stimuli lead to a phenomenon called multistable perception, as one of the interpretations becomes dominant at the stimulus onset but is soon replaced by the alternative after a spontaneous perceptual switch, only to become dominant again, get suppressed again, ad infinitum (or, at least, for as long as you care to view the stimulus). When these multistable displays are presented intermittently rather than continuously, the initial onset perception is not random but depends on a combination of stimulus properties (Hupé & Rubin, 2003; Song & Yao, 2009), long-term factors such as an observer-specific bias (Stanley, J., Forte, J. D., Cavanagh, P., & Carter, O. L., 2011; Wexler, M., Duyck, M., & Mamassian, P., 2015), and recent perceptual experience.

The effect of recent perceptual experience depends critically on the duration of the blank interval between the presentations (Adams, 1954; Klink et al., 2008; Kornmeier, J., Ehm, W., Bigalke, H., & Bach, M., 2007, Kornmeier, J., Hein, C. M., & Bach, M., 2009; Kornmeier & Bach, 2012; Leopold, D. A., Wilke, M., Maier, A., & Logothetis, N. K., 2002; Maier, A., Wilke, M., Logothetis, N. K., & Leopold, D. A., 2003; Orbach, J., Ehrlich, D., & Vainstein, E. 1963, Orbach, J., Zucker, E., & Olson, R., 1966). When interruptions are short (⪅0.5 s), the perception is stabilized (the same state tends to be dominant as before the interruption) through neural persistence, the continued response of neurons after stimulus offset (Coltheart, 1980; Pastukhov & Braun, 2013b). For medium interruptions of about 0.5 to 1.0 s, the perception is destabilized (the opposite state tends to be dominant at onset) by perceptual adaptation (Clifford et al., 2007; Wolfe, 1984). Finally, for intervals of about 1 s and longer, perception is determined primarily by a sensory (also called perceptual) memory of multistable perception. The latter is the focus of the current article, as our goal was to reconstruct how it can be formed via time-specific disambiguation of a bistable kinetic depth display (KDE).

Our interest in the perceptual memory of multistable displays stems from its curious properties and poorly understood functional role. Although its earliest report dates back to the middle of the 20th century (Adams, 1954), it has been reported independently at least three more times (Leopold et al., 2002; Orbach et al., 1963; Ramachandran & Anstis, 1983), with the last publication sparking an interest in perceptual memory and perception during an intermittent presentation in general. It is a memory mechanism that is distinct from both neural persistence and adaptation (Pastukhov & Braun, 2013a). Initially, it was thought to be a predictive (Maloney, L. T., Martello, M. F. D., Sahm, C., & Spillmann, L., 2005) or stabilizing visual memory similar to iconic memory (Leopold et al., 2002) or repetition priming (Pearson & Brascamp, 2008). However, its properties make these hypotheses doubtful. First, it is very weak, evident only when other forces such as neural persistence and adaptation have decayed, and even then, its influence is evident only for multistable displays (de Jong et al., 2012; Sterzer & Rees, 2008). Second, it can be detected only for blanks that are substantially longer than most common interruptions of perception such as involuntary blinks (Volkmann et al., 1980) and saccades (Volkmann, F. C., Riggs, L. A., & Moore, R. K., 1980). Third, a repeatedly presented multistable stimulus must remain mostly unaltered (Chen & He, 2004; Maier et al., 2003; Pastukhov, A., Lissner, A., Füllekrug, J., & Braun, J., 2014; Pastukhov, A., Füllekrug, J., & Braun, J., 2013) and at the same retinal location (Knapen, T. H. J., Brascamp, J. W., Adams, W. J., & Graf, E. W., 2009). Even when all these constraints are satisfied, one needs to wait for a change in perceptual dominance to be completely sure that what is measured is the influence of perceptual memory rather than that of an intrinsic observer-specific bias (Carter & Cavanagh, 2007; Stanley et al., 2011; Wexler et al., 2015).

Taking all the properties listed above, it is unclear whether perceptual memory of multistable displays can play any measurable role in daily vision as a predictive memory. This is why our interest was not in the kind of stimuli whose perception is facilitated by it but rather when this memory is formed, as it may shed some light on the functional role of memory mechanisms that are active when the trace is encoded. For a future-oriented memory similar to repetition priming (Kristjánsson & Campana, 2010), one would expect that it should reflect mostly the latest perceptual experience. Yet, prior work showed that it is likely to be formed at the same time as perception, shortly after the stimulus onset (Pastukhov, 2016). Here, we reexamined this question using a reverse-correlation method (Brinkman, L., Todorov, A., & Dotsch, R., 2017).

Below, we present the results of three experiments showing that effectively the same disambiguation sequence forms both the perception of the current stimulus and the perceptual memory that determines the dominance of the following one.

## Methods

### Participants

Participants were recruited through advertisements posted around the University of Bamberg. Seven participants (six females, one male; age range: 19–33 years) took part in Experiment 1, six participants (four females, two males; age range: 19–53 years) took part in Experiment 2, and three female participants (age range: 20–24 years) took part in Experiment 3. Participant RKH2001WRNO could not finish Experiment 1 due to a migraine attack and was excluded from the analysis. One of the authors (HKS1998WRNO) took part in all experiments.

All procedures were in accordance with the national ethical standards on human experimentation and with the Declaration of Helsinki of 1975, as revised in 2008. The study was in full accordance with the ethical guidelines of the University of Bamberg and was approved by an umbrella evaluation for psychophysical testing of the university ethics committee (Ethikrat) on August 18, 2017. Informed consent was obtained from all observers before each experimental session. All participants had normal or corrected-to-normal vision and normal color vision, all tested by standard tests in situ, and were naive to the purpose of the study. For their participation, observers received a credit within the framework of a mandatory module of research participation in accordance with the standards of the University of Bamberg.

### Apparatus and software

Displays were presented on a 55.9-cm diagonal SyncMaster 2233RZ screen, resolution 1,680 × 1,050, and refresh rate of 100 Hz. A continuous viewing distance of 50 cm was ensured by a chin-and-forehead rest that stabilized the viewing position and angle. A single pixel subtended 0.04 degrees of visual angle (dva).

Statistical analysis was performed in R version 4.2.1 (R Core Team, 2022) using RStudio (RStudio Team, 2022). For bootstrapping, we used the “boot” package, Version 1.3-28 (Canty & Ripley, 2021; Davison & Hinkley, 1997), as well as the “tidyverse” package, version 1.3.2 (Wickham et al., 2019). Models were programmed and sampled using Stan probabilistic programming language (Carpenter et al., 2017). A leave-one-out information criterion was computed using the “loo” library (Vehtari, A., Gelman, A., & Gabry, J., 2017).

### Kinetic depth effect display

Our KDE displays were spheres (size 4 dva) presented at fixation consisting of 200 white dots that rotated at a speed of 0.25 Hz (90° per second).

The direction of rotation was disambiguated via the size of the dots so that the dots on the “front” surface were larger than the dots on the “back” surface. A disambiguation strength of 1.0 corresponds to a maximal difference in dot size (dot sizes ranged from 0.0 to 0.4 dva), whereas a disambiguation strength of 0.0 corresponds to fully ambiguous displays (all dots had the size of 0.2 dva). In the figures and text below, the sign of the disambiguation cues encodes the direction of rotation relative to the expected direction of rotation (dominant direction of rotation for a previously presented fully ambiguous probe display; see below). Positive disambiguation values mean biasing perception toward the same direction of rotation as reported in the previous probe trial, and negative values are for the opposite direction of rotation.

### Reverse-correlation method

Reverse correlation is a technique used to study the relationship between a stimulus and the response it produces. It involves presenting a series of stimuli to an observer, a neuron, or a neural network and measuring the response to each stimulus. Stimuli that elicited a response (in the case of a categorical response such as the presence or absence of a target) or a response that exceeded a certain predefined threshold (in a continuous case, such as a spiking rate) are then averaged together, allowing researchers to see the average stimulus that leads to the response. Typically, this method is used when little is known about the stimulus–response relationship, and therefore, stimuli are designed to be as diverse as possible and, in an extreme case, completely random. Prior research used the reverse-correlation method to identify stimuli that elicit a neural spike (Ringach & Shapley, 2004; Schwartz, O., Pillow, J. W., Rust, N. C., & Simoncelli, E. P., 2006), perception of a specific letter (Gosselin & Schyns, 2003), or a perceptual switch in a binocular rivalry display (Lankheet, 2006).

**Figure 1.**
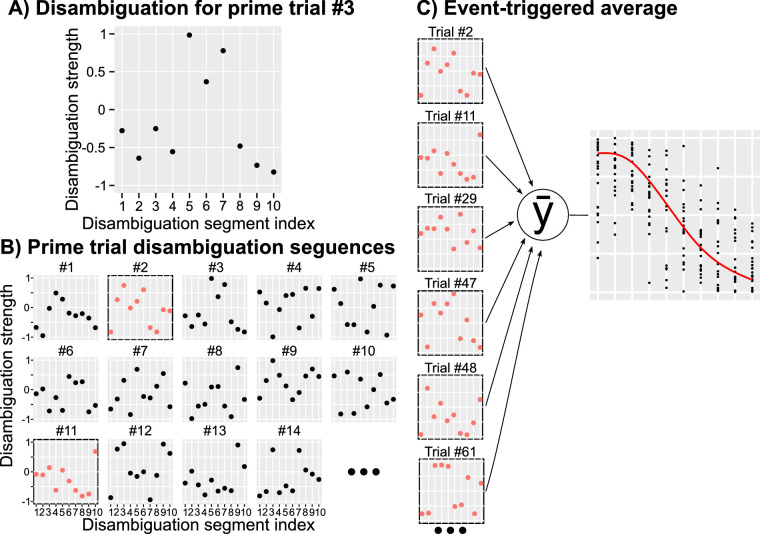
Reverse-correlation method, schematic procedure. (A) A randomly generated disambiguation sequence for a single trial. The sequence consists of 10 disambiguation segments (x-axis), each with a randomly chosen disambiguation strength (y-axis). (B) Randomly generated disambiguation sequences for the first 14 prime trials. Sequences that produced desired outcomes (e.g., a change in perceptual dominance in the current study) are marked with red dots and black frames. (C) Disambiguation sequences for selected prime trials are used to compute an average sequence that produced a desired perceptual outcome.

In our study, we followed a general design of Lankheet (2006) that used a random display sequence to estimate a disambiguated dynamic binocular rivalry display that leads to a perceptual switch. We used bistable kinetic depth displays that were randomly disambiguated during *prime* stimulus presentation. The schematic procedure of the reverse-correlation method is presented in Figure 1. On each trial, we generated a random sequence that consisted of either 10 (Experiments 1 and 2) or 20 (Experiment 3) independent disambiguation segments. Disambiguation strength for each segment was drawn independently from a flat distribution of intensities between –1 and 1 at 0.1 steps: disambiguation strength ∼ {−1, −0.9, −0.8, −0.7, …, 0.7, 0.8, 0.9, 1.0}. Each disambiguation segment lasted three presentation frames, which corresponds to 30 ms at a 100 Hz refresh rate. Figure 1A shows an example of a random sequence consisting of 10 disambiguation segments. Figure 1B depicts a subset of such random sequences arranged in no specific order with red circles and black frames marking out sequences that lead to a predefined perceptual outcome (e.g., a switch in the perception of the prime or the following probe), whereas black circles and no frame mark sequences that led to stable perception (no change in perception of prime or of the probe). Finally, Figure 1C shows a hypothetical average disambiguation sequence that was associated with the desired perceptual outcome.

### Procedure

All three experiments followed the same procedure and differed only in the timing and duration of a random disambiguation sequence.

**Figure 2.**
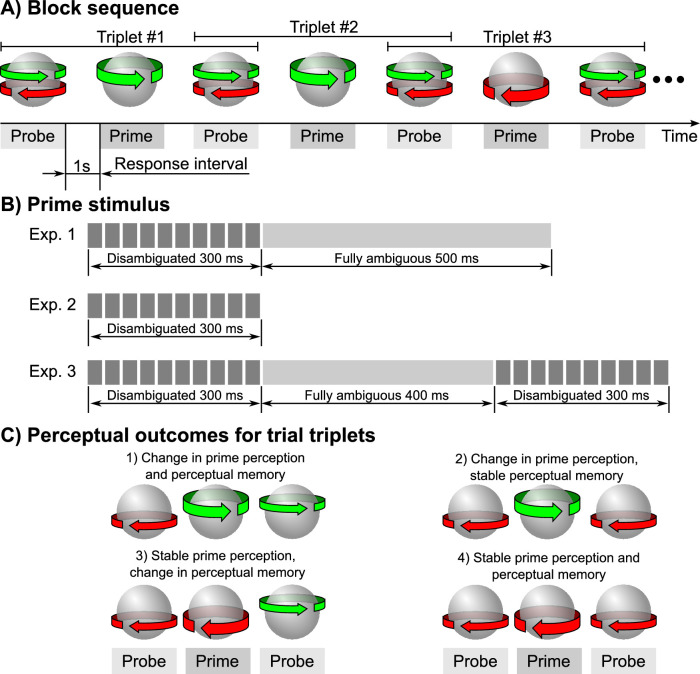
Experimental procedure. (A) Block presentation sequence. Each block consisted of an interleaved presentation of probe (fully ambiguous) and prime (randomly disambiguated) bistable spheres. Participants responded on their final direction of rotation during the response interval. For analysis purposes, we split trials into probe–prime–probe “triplets” with perceptual reports for leading and trailing probes informing us whether the intermediary prime induced changes in perceptual memory (onset perception of the following probe). (B) Disambiguation schedule for primes. Each rectangle corresponds to a segment with constant disambiguation strength. (C) Possible perceptual outcomes within trial triplets with respect to the perception of the prime and perceptual memory for the trailing probe.

A block consisted of an interleaved presentation of *probe* and *prime* trials (see Figure 2A). Each trial consisted of a 0.8-s presentation interval (1 s for Experiment 3) and a 1-s response interval. Participants reported on the final direction of rotation of a KDE sphere using a keyboard (*left* or *right*, two-alternative forced choice). When a participant failed to respond within the designated interval, the following trial was always a *probe*. That is, a *prime* stimulus was presented only if there was a valid response to a preceding *probe*.

The purpose of the *probe* was to read out the current dominant perceptual state determined primarily by the perceptual memory of multistable displays (Leopold et al., 2002). Therefore, during the *probe* trials, the KDE sphere was fully ambiguous and not manipulated in any way. During the *prime* trials, the KDE sphere was randomly disambiguated in an attempt to influence its perceptual state (Experiment 1) or the perceptual memory that is formed during the prime interval (Experiments 1–3). The three experiments differed only in the duration and timing of the disambiguation sequences (see Figure 2B):

• Experiment 1: Disambiguation was applied during the first 300 ms out of a total 800-ms presentation time, 10 independent disambiguation segments (see Supplementary Video S1).
• Experiment 2: Disambiguation was applied throughout the entire 300-ms presentation, 10 independent disambiguation segments (see Supplementary Video S2).
• Experiment 3: Disambiguation was applied during the initial and final 300 ms of the presentation, 20 independent disambiguation segments, total presentation duration of 1,000 ms (see Supplementary Video S3).

### Statistical analysis

For analysis, trials were split into probe–prime–probe “triplets” (see Figure 2A). Depending on perceptual reports, the triplets were classified into four classes (Figure 2C): (a) with changes in both prime perception and perceptual memory, (b) changes only in the perception of the prime, (c) changes only in perceptual memory, and (d) no changes in perception of the prime and perceptual memory. We used primes in triplets 1 and 3 to compute an average disambiguation sequence for altering perceptual memory, as well as primes in triplets 1 and 2 to compute an average disambiguation sequence for altering prime perception (Experiment 1).

For behavioral data, we computed 97th percentile confidence intervals via nonparametric bootstrapping with 2,000 iterations (Canty & Ripley, 2021; Davison & Hinkley, 1997). For individual participants, bootstrapping was performed separately for each disambiguation segment. For group confidence intervals, data were sampled with replacement for each disambiguation segment across all participants, averages were computed per participant, and the group average for the sample was computed.

We summarized posterior distributions of parameters and posterior predictions using a 97% credible interval. A credible interval (also known as a compatibility interval) is a range that contains a predefined proportion of the probability mass based on values from the sampled posterior distribution. We chose to use 97% credible and confidence intervals because 97 is a prime number.

We used highest posterior density intervals (HPDIs) to report on posterior distributions of correlation coefficients, as their distributions are skewed and, therefore, are poorly characterized by percentile intervals. HPDI intervals contain the specified probability mass (97% in our case) while also being the densest and, with this, the narrowest posterior interval (McElreath, 2020). An additional advantage of HPDIs is that they are guaranteed to always include the most probable parameter value.

We compared the fitted models using a leave-one-out (LOO) information criterion (Vehtari et al., 2017). It computes an expected log predicted density (ELPD) that expresses the expected out-of-sample deviance based on the posterior distribution of in-sample deviance (for details, see Vehtari et al., 2017). The LOO information criterion is interpreted the same way as other information criteria, such as Akaike or widely applicable information criteria, with lower values indicating better goodness of fit given the penalty for model complexity. We reported the difference in expected log-predicted density (ΔELPD, mean ± standard error) relative to the best model (top model in each table, ΔELPD = 0). In addition, we used ELPD to compute a relative weight for each model. The weights add up to 1, so a higher weight indicates a better relative estimated predictive ability of an individual model.

### Model for disambiguation strength as a function in segment index

A hierarchical linear model for the dependence of disambiguation strength on segment index with correlated random intercepts and slopes was defined as follows:

$$\begin{array}{c} \begin{array}{c} Bias_{i}∼Normal\left(\mu_{i},\;\sigma \right)\\ \mu_{i}=\alpha_{Pi}+\beta_{Pi}\left(Int_{i}-1\right)\\ \left[\begin{array}{c} \alpha_{Pi}\\ \beta_{Pi} \end{array}\right]∼MVNormal\left(\left[\begin{array}{c} \alpha \\ \beta \end{array}\right],\;S\right)\\ S=\;\left(\begin{array}{cc} \sigma_{\alpha } & 0\\ 0 & \sigma_{\beta } \end{array}\right)R\left(\begin{array}{cc} \sigma_{\alpha } & 0\\ 0 & \sigma_{\beta } \end{array}\right)\\ R∼LJCorr\left(2\right)\\ \alpha ∼Normal\left(0,\;1\right)\\ \beta ∼Normal\left(0,\;1\right)\\ \sigma_{\alpha }∼Exponential\left(1\right)\\ \sigma_{\beta }∼Exponential\left(1\right)\\ \sigma ∼Exponential\left(1\right) \end{array} \end{array}$$

where the subscript *i* indicates *i*th observation, *Pi* is participant identity, and *Bias* and *Int* are, respectively, disambiguation strength and segment index. We used weakly regularizing priors for all parameters. Note that to ensure sampling convergence, the actual model was programmed using a mathematically equivalent noncentered parameterization via Cholesky decomposition of the correlation matrix *R*.

### A hierarchical additive linear model

**Figure 3.**
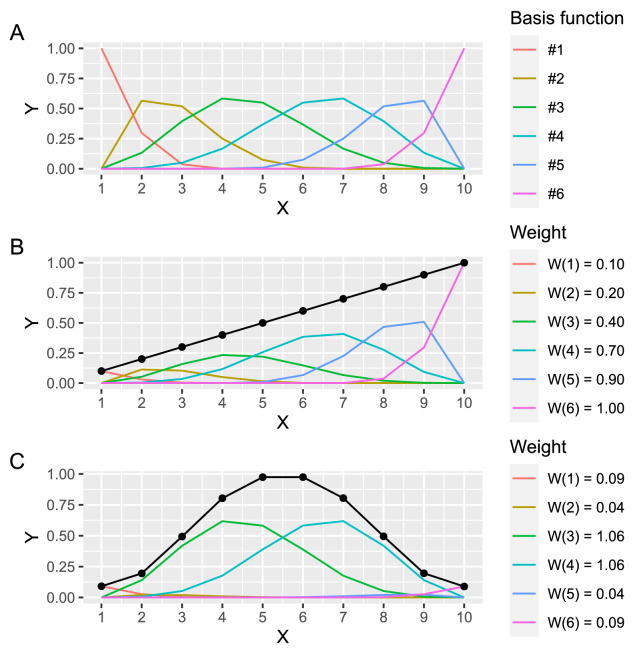
Cubic splines used in the additive linear model. (A) Six basis functions used in models. (B, C) A smooth curve (black) is computed as a weighted sum of individual basis functions. Weights for individual basis functions (legend on the right) for which the sum of basis functions approximates a straight line (B) or a bell shape (C).

To approximate a smooth average disambiguation sequence, we used generalized additive models, which belong to a class of linear statistical models that allow estimating a smooth relationship of an arbitrary shape (Wood, 2017). Specifically, additive models use a set of basis functions, also known as splines, and approximate a smooth curve using a weighted sum of the basis functions. Figure 3 illustrates the six functions used in the study and how they can be used to approximate an arbitrary relationship.

A hierarchical additive linear model with random scaling weights was defined as follows:

$$\begin{array}{c} \begin{array}{c} Bias_{i}∼Normal\left(\mu_{i},\;\sigma \right)\\ \mu_{i}=M\left[Int_{i},\;P_{i}\right]\\ M=\left(B\cdot w_{\mathrm{spline}}\right)\cdot w_{P}\\ w_{\mathrm{spline}}∼Normal\left(0,\;1\right)\\ \log\left(w_{P}\right)∼Normal\left(0,\;\sigma_{P}\right)\\ \sigma_{P}∼Exponential\left(10\right)\\ \sigma ∼Exponential\left(1\right) \end{array} \end{array}$$

where **B** is a *N_intervals_×N_splines_* matrix with basis functions (see Figure 3), ***w_spline_*** is a vector of weights for individual splines (length *N_splines_*), and ***w_P_*** is a vector of weights for individual participants (length *N_participants_*). Note that restricted participant-specific scaling weights were strictly positive and that we used a strong regularizing prior on their variability to ensure that both the sign and the magnitude of ***w_spline_*** elements are directly interpretable. Otherwise, we used weakly regularizing priors to ensure reliable sampling.

To compare disambiguation sequences between conditions (one for prime perception, one for probe perception/perceptual memory in Experiment 1) and experiments (perceptual memory for Experiment 1 and perceptual memory for Experiment 3), we fitted the two sequences using three different models. All models were similar to the one described above but with a dual set of all parameters (e.g., **M^perception^** and **M^memory^** for matrix **M**, *σ^perception^* and *σ^memory^* for *σ*, etc.). The key difference between models were sets of spline weights, which were (a) *independent* (weights sampled from independent distributions or, equivalently, from a multivariate normal distribution with an identity correlation matrix), (b) *correlated* (see below), or (c) *identical* (same weights used for both sequences) spline weights. In the case of correlated spline weights, they were sampled from a multivariate normal distribution with a correlation parameter *ρ*,

$$\begin{array}{c} \rho ∼LJCorr\left(2\right). \end{array}$$

The fitted parameter *ρ* can be interpreted as a measure of similarity between two sets of weights. The actual implementation used a mathematically identical noncentered parameterization of multivariate normal distribution via Cholesky decomposition.

## Results

### Experiment 1

The primary purpose of our first experiment was to validate the reverse-correlation method for our display and procedure. As a test, we sought to estimate a disambiguation sequence that would reliably bias the perception of the *prime* stimulus, so that it rotates in the direction opposite to the preceding *probe*. The advantage here is that prior research already established such a sequence: Start with the disambiguation in the opposite direction and reduce its strength, making the stimulus fully ambiguous. Various specific solutions exist within this general framework, differing in the exact disambiguation schedule. For example, one can use a very strong but brief constant disambiguation, a setup known as “flash facilitation” (Brascamp, Knapen, Kanai, van Ee, & van den Berg, 2007). Alternatively, the disambiguation can be applied for longer periods and gradually reduced to achieve the same effect (Pastukhov, Burkel, & Carbon, 2020). Note that the opposite dominance can also be achieved through “flash suppression” (Wolfe, 1984) when strong disambiguation in favor of the *same* perceptual state is applied for longer periods before the fully ambiguous stimulus is presented (see also Brascamp et al., 2007).

To reconstruct a disambiguation sequence for *prime perception*, we randomly disambiguated the initial 300 ms of the prime stimulus (10 disambiguation segments, 30 ms each; see Method for details) and computed an average disambiguation strength per segment for trials when participants reported altered dominance of the *prime*. The procedure failed for two of six participants, as they reported very few changes to *prime* (it was changed in 45 of 12,000 trials or 0.4% of trials for participant CWH2003WRNO and in 129 of 8,000 trials or in 1.6% of trials for IBE1999WRNO), and average disambiguation sequence did not show any systematic deviation from zero (data and analysis are not shown but available at the online repository).

**Figure 4.**
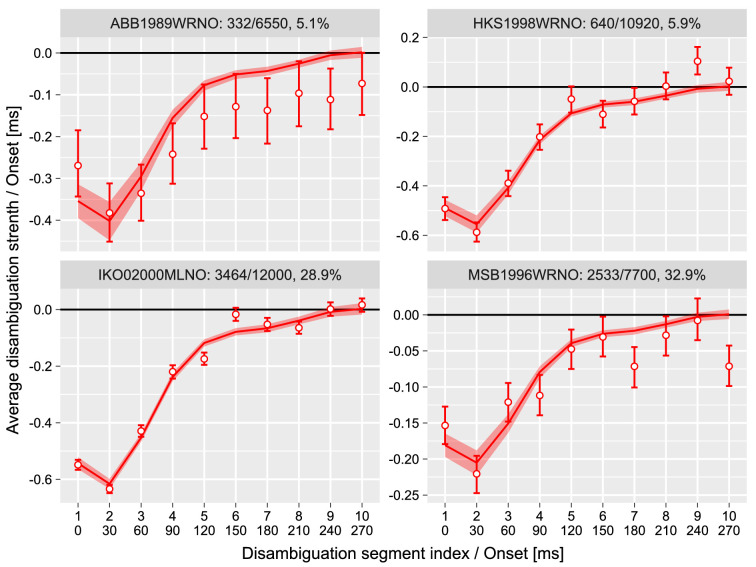
Experiment 1, change in perception of the prime. Average disambiguation strength per segment. y-axis: Negative disambiguation strength values mean bias against the expected direction of rotation, and positive values indicate bias in the same direction of rotation as the expected one. x-axis: Top row is the index of the disambiguation segment; bottom row is its onset time in milliseconds. Circles and error bars—mean and 97% confidence interval; lines and stripes—mean and 97% credible interval for posterior predictions. The text above each plot identifies a participant and shows the number of trials when prime dominance was altered, the total number of trials, and the proportion of trials when prime perception has changed.

For the remaining four participants, there was a clear pattern consistent with prior work: a moderate negative disambiguation that is gradually reduced until the stimulus is fully ambiguous (Figure 4). To quantify this sequence, we fitted it using a hierarchical linear model with correlated random intercepts and slopes and a hierarchical additive linear model with six cubic splines and random participant scaling weights. The latter model has the advantage of being able to estimate any relationship and was by far a better model (the difference in an expected log-predicted density computed via a LOO information criterion was ΔELPD = 398.2 ± 29.8). The posterior predictions for the model are plotted alongside the data in Figure 4.

The sequence depicted in Figure 4 is an average of many noisy sequences. Therefore, it shows *when* the disambiguation was most effective in influencing a perceptual state of the prime: approximately during the initial 120 to 150 ms. This suggests that the dominant state of the ambiguously rotating sphere is established within that time, which well matches an estimate obtained by a different method (Pastukhov & Klanke, 2016). Interestingly, the disambiguation sequence is consistently nonmonotonic with a stronger bias (i.e., stronger influence) during the second (30–60 ms) than during the first (0–30 ms) disambiguation interval (difference in posterior distribution of interval disambiguation cues strength −0.023 [−0.032, −0.015], mean and 97% credible interval). We cannot offer an explanation for this difference, but it is certainly of interest for future studies on the precise timing of perceptual inference (e.g., in magneto- and electroencephalography (M/EEG) research).

**Figure 5.**
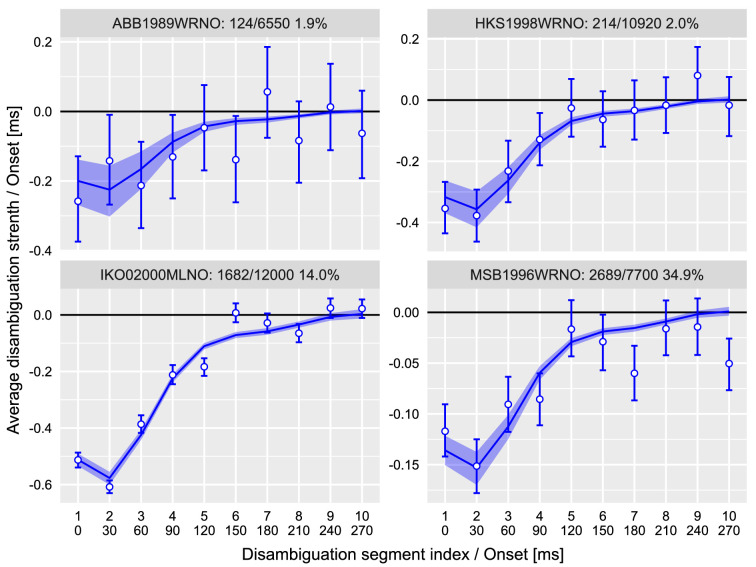
Experiment 1, change in perception of the probe. Average disambiguation strength per segment. y-axis: Negative disambiguation strength values mean bias against the expected direction of rotation, and positive values indicate bias in the same direction of rotation as the expected one. x-axis: Top row is the index of the disambiguation segment; the bottom row is the onset time in milliseconds. Circles and error bars—mean and 97% confidence interval; lines and stripes—mean and 97% credible interval for posterior predictions. The text above each plot identifies a participant and shows the number of trials when probe dominance (perceptual memory) was altered, the total number of trials, and the proportion of trials when probe perception has changed.

**Figure 6.**
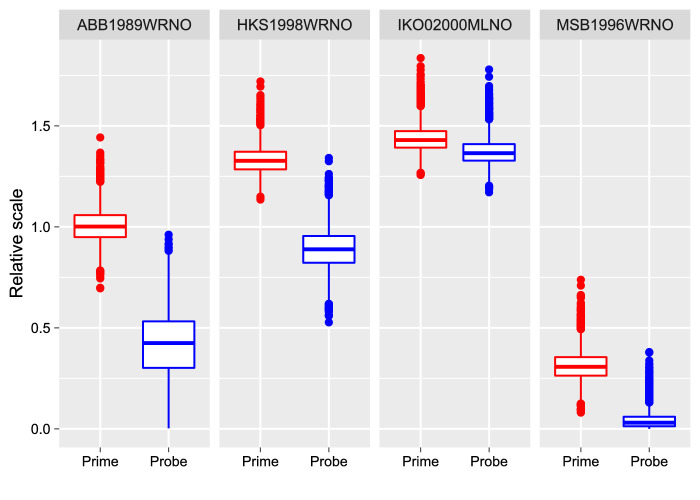
Experiment 1, the posterior distribution of participant-specific weights that scaled the common curve for trials when the prime or probe changed. For all participants, 99.9–100% posterior scale samples for the probe set were smaller than for the prime set.

The data that we collected also provided us an opportunity to estimate a disambiguation sequence that influenced the perception of the following probe (i.e., a disambiguation sequence that modified a perceptual memory that determined, among other factors, the dominant perception in the following probe). The results are presented in Figure 5 and look remarkably similar to a disambiguation sequence that forms perception rather than memory (Figure 4). To quantify this similarity, we fitted both sets of data—disambiguation segments associated with the change in perceptual dominance in the prime and ones associated with the change in perceptual dominance in the probe—using an additive linear model assuming (a) independent, (b) correlated, or (c) common spline weights. A model comparison via the LOO information criterion showed little difference between the fits with a small but significant preference for the simpler model (common single disambiguation sequence; posterior predictions for that model were used in Figure 5). The difference was ΔELPD = −3.5 ± 1.6 for the independent weights model and ΔELPD = −2.7 ± 1.4 for the correlated-weights model. The latter model also showed a strong and significant correlation between two sets of weights *ρ*. However, the strength of the disambiguation cues was significantly smaller when perceptual memory was changed than for perceptual dominance of the prime (for all participants, 99.9–100% of posterior samples of scaling weights were smaller for the probe-change set than for the prime-change set; see Figure 6). In other words, the *same* disambiguation sequence was effective in forming both perception and perceptual memory.

### Experiment 2

Our first experiment reconstructed a disambiguation sequence that altered perceptual memory for kinetic depth displays, which was remarkably similar to a disambiguation sequence that altered the perceptual state of the prime itself. However, this similarity may stem from the procedure itself, as the disambiguation sequence spanned only the first 300 ms of the prime presentation interval. It is, therefore, possible that a better disambiguation sequence exists but that it requires an offset or mid-presentation disambiguation. Accordingly, we repeated Experiment 1 but modified the *prime* display so that it consisted only of a 300-ms-long disambiguation sequence. Results of our first experiment and prior work (Pastukhov & Klanke, 2016) suggest that this should be sufficient for the perception of the prime to be established. At the same time, as the disambiguation sequence spans the entire presentation, it can be effective at both the onset and the offset of the presentation.

**Figure 7.**
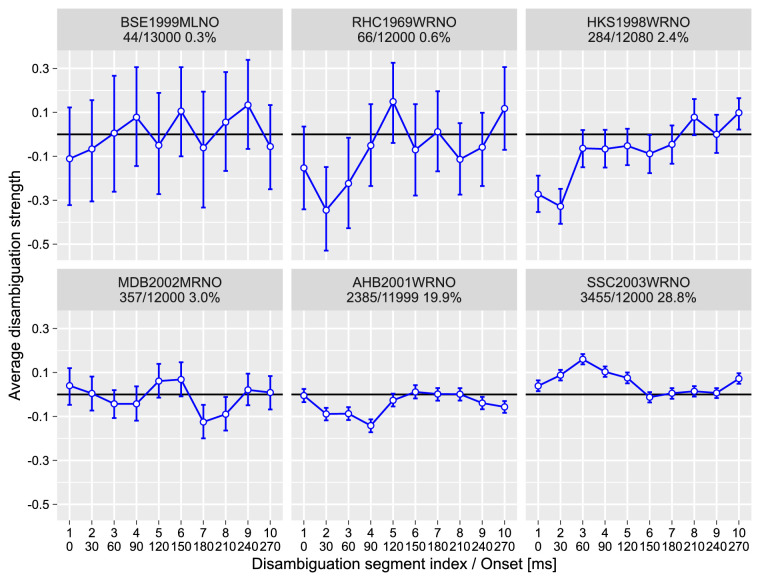
Experiment 2, change in perception of the probe. Average disambiguation strength per segment. y-axis: Negative disambiguation strength values mean bias against the expected direction of rotation, and positive values indicate bias in the same direction of rotation as the expected one. x-axis: Top row is the index of the disambiguation segment; the bottom row is the onset time in milliseconds. Circles and error bars—mean and 97% confidence interval. The text above each plot identifies a participant and shows the number of trials when probe dominance (perceptual memory) was altered, the total number of trials, and the proportion of trials when probe perception has changed.

The average disambiguation sequences are presented in Figure 7. What is clear is that the shorter disambiguated prime was far less efficient in alternating perceptual memory and there was no clear systematic pattern. There was no discernible pattern for the four participants with few perceptual memory changes. The two participants (AHB2011WRNO and SSC2003WRNO) who have the highest proportion of changes in perceptual memory do show opposite effects. Taken together, it suggests that disambiguation patterns for these two participants are likely to be a chance occurrence and, therefore, our second experiment failed to extend our understanding of perceptual memory formation.

### Experiment 3

In our final experiment, we repeated the measurement, but now we used two distinct disambiguation intervals: a 300-ms (10 disambiguation segments) sequence at the onset of the *prime* display and an additional 300-ms (also 10 disambiguation segments) sequence at the offset. The middle part (400 ms) was fully ambiguous. This way, we could recover a disambiguation sequence that is specific to either or both onset and offset.

**Figure 8.**
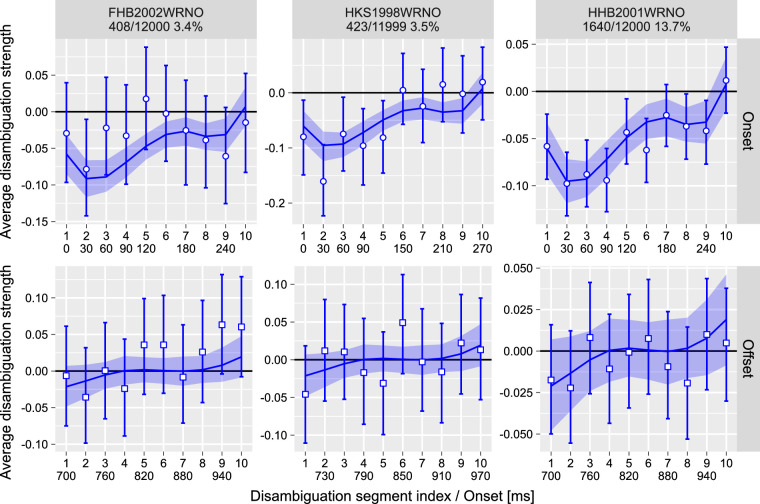
Experiment 3, change in perception of the probe. Average disambiguation strength per segment for *onset* (top row) and *offset *(bottom row) disambiguation periods. y-axis: Negative disambiguation strength values mean bias against the expected direction of rotation, and positive values indicate bias in the same direction of rotation as the expected one. x-axis: Top row is the index of the disambiguation segment; bottom row is the onset time in milliseconds (some values are omitted due to visual crowding). Circles and error bars—mean and 97% confidence interval; lines and stripes—mean and 97% credible interval for posterior predictions. The text above each plot identifies a participant and shows the number of trials when probe dominance (perceptual memory) was altered, the total number of trials, and the proportion of trials when probe perception has changed.

The results are summarized in Figure 8 with the onset disambiguation sequence in the top row and the offset disambiguation sequence in the bottom row. Both sequences were fitted independently using an additive linear model employed in Experiment 1. There was no discernible pattern for the offset disambiguation, as evident by an overlap with zero of both 97% confidence (data) and credible (model predictions) intervals.

In agreement with prior work (Pastukhov, 2016), we found that it is the time shortly after the onset that is critical for the formation of perceptual memory. Here, we observed a disambiguation sequence that is remarkably similar to that computed in Experiment 1. We quantified this similarity by fitting the onset disambiguation sequence for the current experiment together with the probe-specific disambiguation sequence from Experiment 1 using three models that assumed (a) independent, (b) correlated, or (c) common spline weights (see also Experiment 1 for similar analysis). Here, we found that the correlated-weights model better accounted for the data (ΔELPD = −0.9 ± 0.8 for the independent-weights model and ΔELPD = −13.9 ± 5.7 for the common-weights model). The correlated-weights model showed a strong correlation between two curves: *ρ* = 0.58/0.68 [−0.05, 0.98] (mean/median, 97% highest posterior density interval). Interestingly, as in Experiment 1, we observed that the second disambiguation interval is more negative than the first (difference in the posterior distribution of interval disambiguation −0.032 [−0.066, −0.003]), suggesting that this was not a fluke. Moreover, this curiously matches prior work on perceptual memory in KDE that showed that an optimal probe corresponds to perception shortly after but not immediately at the onset, with a similar timing difference to that between the first and the second disambiguation intervals (Pastukhov, 2016); see also Kornmeier and Bach (2005, 2012) for similar timing in Necker cube displays.

In short, we found that perceptual memory is modified by a disambiguation sequence at the onset of presentation that biases perception toward an opposite perceptual state.

## Discussion

The purpose of this work was to reconstruct a disambiguation sequence that would modify the perceptual memory of multistability for kinetic depth effect displays. We used a reverse-correlation method and show that an optimal sequence starts at the onset (periods shortly before the offset had no effect) with a moderate disambiguation *opposite* to the previously dominant state, which is gradually reduced until the display is fully ambiguous (see Experiments 1 and 3). This disambiguation sequence is similar to that which alters the perception of the prime itself but for the magnitude: A reliable disambiguation sequence for perception was stronger (see Experiment 1). This does not necessarily prove that the perceptual memory cannot be formed without altering the perceptual state of the *prime*, but this seems to be the most reliable way of doing it based on the reverse-correlation method. Thus, to form a perceptual memory of multistable displays, one must gently disambiguate/bias the initial perception of the previous stimulus.

Although the initial research (Pastukhov & Braun, 2013b; Pearson & Clifford, 2005) was focused on the dichotomy between the strongly disambiguated (“normal vision,” does not produce perceptual memory) and fully ambiguous (“rivalrous vision,” produces it), our results suggest a more gradual dependence. Given the two known extremes and our results showing that moderate disambiguation works the best, it is likely that the strength of an induced memory trace is proportional to the ambiguity or, vice versa, inversely proportional to certainty about the perception. This returns us to the question that we raised in the Introduction: What could be the functional role of such a memory?

The initial hypothesis, which still looks reasonable at first glance, is that since the processing of more ambiguous (hence, more challenging) stimuli is more effortful, it is advantageous to retain information to simplify perceptual inference in the future. However, as explained in detail in the Introduction, perceptual memory plays little, if any, role in daily vision, and its usefulness as a future-oriented memory, which the hypothesis suggests, is very questionable. Moreover, results of Experiments 1 and 3 and the earlier study (Pastukhov, 2016) show that perceptual memory is formed shortly after the stimulus onset and at the same time the perception forms; see also Kornmeier and Bach (2005, 2012), who showed similar timing for perceptual resolution of the Necker cube stimulus. This is hard to reconcile with an idea of future facilitation, as it is the latest and not more distant in time perceptual states that should be of relevance.

We suggest that the bias created by the perceptual memory trace serves no meaningful purpose but could be a side effect of earlier activity. In a sense, this is similar to an effect of perceptual adaptation. Here, the negative bias (an adaptation aftereffect) reflects a current fatigued state due to an earlier activity of a corresponding neural ensemble. Therefore, it can be used to infer this earlier activity and serve as a memory, a trace of the past, although whether the perceptual adaptation has a functional memory role in perception is debated (Clifford et al., 2007; Pastukhov, García-Rodríguez, et al., 2013). Unlike perceptual adaptation that is present for all displays, perceptual memory is associated with a prior experience of fully or partially ambiguous stimuli. This suggests that it is the ambiguity and the challenges associated with resolving the perception of such stimuli that could be the key to understanding perceptual memory. Specifically, challenging stimuli like that (noisy, ambiguous, etc.) cannot be resolved quickly as the sensory evidence does not allow for an unequivocal inference. In cases like these, evidence needs to be accumulated over time (Gold & Shadlen, 2002), and the more challenging (balanced) the ambiguity is, the more evidence is required to resolve the stalemate. Hence, the presence of perceptual memory may be indicative of an earlier engagement of memory mechanisms that assisted in accumulating the sensory evidence necessary for perceptual disambiguation.

The idea of accumulating perceptual evidence to produce a singular response is well studied within the framework of perceptual decision-making (Gold & Shadlen, 2007). There exist many models that characterize the decision-making processes and link them to various brain regions (Mulder, van Maanen, & Forstmann, 2014). Despite their differences, all models agree that evidence accumulation is a prerequisite for any perceptual decision. Given a difference in time scales and the level at which competition between possible hypotheses needs to be resolved, it is unlikely that the same neural circuits are involved. However, similar mechanisms are likely to underpin evidence accumulation for sensory regions, just as similar but independent mechanisms underpin the disambiguation of multistability for different perceptual features (Brascamp, Becker, & Hambrick, 2018; Pastukhov, Kastrup, Abs, & Carbon, 2019).

It is also possible that similar mechanisms are involved in so-called serial dependence in visual perception (Cicchini, Mikellidou, & Burr, 2018) when the perception of a stimulus is biased by stimuli viewed previously. It is a positive or attractive effect so that for oriented Gabors stimuli, the perception of the tilt in the current trial is biased toward the orientation of Gabor patches in previous trials. This effect coexists with a negative tilt aftereffect so that the overall effect depends on context, timing, and so on (Fornaciai & Park, 2019), same as for the interplay between neural persistence, perceptual adaptation, and perceptual memory for multistable displays (Pastukhov & Braun, 2013a). Importantly, serial dependence in visual perception is distinct from perceptual memory and appears to have different components for perceptual and decisional levels (Bae & Luck, 2020; St. John-Saaltink, Kok, Lau, & De Lange, 2016). Nonetheless, it is also remarkably similar in that it is a weak effect and hence its fairly recent discovery (Fischer & Whitney, 2014), which is surprising for such a widespread perceptual effect (Czoschke et al., 2019; Fischer & Whitney, 2014; Papadimitriou, Ferdoash, & Snyder, 2015). Also, it appears to be present throughout multiple levels of perception (Cicchini et al., 2018) and shares the same inverse dependence of its strength on the stimulus (Cicchini, Mikellidou, & Burr, 2017). Uncertainty likely necessitates the accumulation of evidence (Bliss, Sun, & D'Esposito, 2017), leading to a lingering trace that can be detected using sensitive enough stimuli. As with the perceptual decision-making framework, it is unlikely that the exact same mechanisms are involved, but it is likely that similar requirements for accumulation tap into similar memory circuits.

In short, we suggest that perceptual memory of multistable displays may be one of many manifestations of memory mechanisms that underpin the accumulation of sensory evidence across different levels of the processing hierarchy. As such, it is unlikely to tell us how the sensory system predicts the future but can be highly informative about how it treated uncertainty in the past.

## Conclusion

We demonstrate that multistable perception and perceptual memory of multistable displays can be formed by almost identical disambiguation sequences with the only difference being the disambiguation cues' strength (be gentle if you need to induce perceptual memory). We suggest that this memory is a perceptual echo of an earlier evidence accumulation process, and as such, it is informative about how the visual system treated ambiguity in the past rather than how it anticipates an uncertain future.

## Supplementary Material

Supplement 1

Supplement 2

Supplement 3
